# 3-(2-Amino-1-methyl-4-oxo-4,5-dihydro-1*H*-imidazol-5-yl)-3-hydroxy­indolin-2-one monohydrate

**DOI:** 10.1107/S1600536809004875

**Published:** 2009-02-21

**Authors:** Narsimha Reddy Penthala, Thirupathi Reddy Yerram Reddy, Sean Parkin, Peter A. Crooks

**Affiliations:** a Department of Pharmaceutical Sciences, College of Pharmacy, University of Kentucky, Lexington, KY 40536, USA; b Department of Chemistry, University of Kentucky, Lexington, KY 40506, USA

## Abstract

Two chiral centres exist in the title compound, C_12_H_12_N_4_O_3_·H_2_O. Mol­ecules are linked into chains by series of inter­molecular N—H⋯O and O—H⋯N hydrogen bonds, which causes supra­molecular aggregation. Two chiral centres are formed in the title compound. The indole and creatinine moieties make a dihedral angle of 56.75 (4)°. The crystal structure of the compound indicates the presence of equimolar enantio­mers (*RR* and *SS*) in the crystal structure.

## Related literature

For 2-indol-3-yl-methyl­enequinuclidin-3-ols NADPH oxidase activity, see: Sekhar *et al.* (2003 ▶). For novel substituted (*Z*)-2-(*N*-benzyl­indol-3-ylmethyl­ene)quinuclidin-3-one and (*Z*)-(±)-2-(*N*-benzyl­indol-3-ylmethyl­ene)quinuclidin-3-ol derivatives as potent thermal sensitizing agents, see: Sonar *et al.* (2007 ▶). For the crystal and mol­ecular structure of isatin, see: Frolova *et al.* (1988 ▶). For the structure of 1,1′-diacetyl-3-hydr­oxy-2,2′,3,3′-tetra­hydro-3,3′-bi(1*H*-indole)-2,2′-dione, see: Usman *et al.* (2002 ▶). The aldol condensation enolate mechanism by six-membered transition states has been described by Zimmerman & Traxler (1957 ▶).
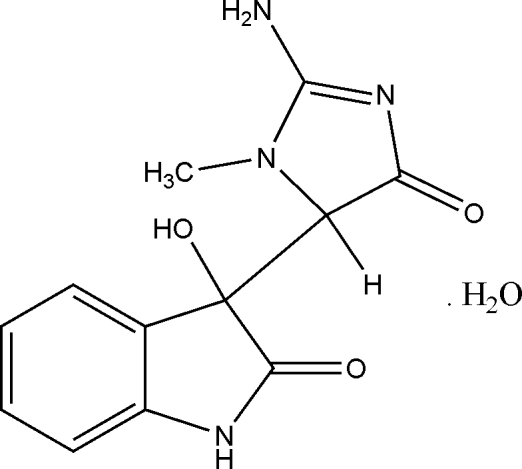


## Experimental

### 

#### Crystal data

C_12_H_12_N_4_O_3_·H_2_O
*M* *_r_* = 278.27
Monoclinic, 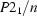

*a* = 8.3514 (1) Å
*b* = 10.7166 (2) Å
*c* = 13.9679 (2) Å
β = 104.755 (1)°
*V* = 1208.88 (3) Å^3^
*Z* = 4
Cu *K*α radiation
μ = 0.99 mm^−1^
*T* = 90 K
0.20 × 0.15 × 0.06 mm

#### Data collection

Bruker X8 Proteum diffractometer
Absorption correction: multi-scan (*SADABS*; Bruker, 2006 ▶) *T*_min_ = 0.780, *T*_max_ = 0.943
17255 measured reflections
2181 independent reflections
2121 reflections with *I* > 2σ(*I*)
*R*_int_ = 0.035

#### Refinement

*R*[*F* ^2^ > 2σ(*F* ^2^)] = 0.034
*wR*(*F* ^2^) = 0.088
*S* = 1.04
2181 reflections
191 parameters
3 restraints
H atoms treated by a mixture of independent and constrained refinement
Δρ_max_ = 0.26 e Å^−3^
Δρ_min_ = −0.27 e Å^−3^

### 

Data collection: *APEX2* (Bruker, 2006 ▶); cell refinement: *APEX2* and *SAINT* (Bruker, 2006 ▶); data reduction: *SAINT*; program(s) used to solve structure: *SHELXS97* (Sheldrick, 2008 ▶); program(s) used to refine structure: *SHELXL97* (Sheldrick, 2008 ▶); molecular graphics: *XP* in *SHELXTL* (Sheldrick, 2008 ▶); software used to prepare material for publication: *SHELXL97* and local procedures.

## Supplementary Material

Crystal structure: contains datablocks global, I. DOI: 10.1107/S1600536809004875/hg2475sup1.cif

Structure factors: contains datablocks I. DOI: 10.1107/S1600536809004875/hg2475Isup2.hkl

Additional supplementary materials:  crystallographic information; 3D view; checkCIF report

## Figures and Tables

**Table 1:** Hydrogen-bond geometry (Å, °)

*D*—H⋯*A*	*D*—H	H⋯*A*	*D*⋯*A*	*D*—H⋯*A*
N1—H1⋯O1*W*^i^	0.88	1.96	2.8128 (15)	163
O8—H8⋯N12^ii^	0.84	1.97	2.7984 (14)	170
N11—H11*A*⋯O13^iii^	0.88	2.17	3.0371 (15)	171
N11—H11*B*⋯O1^iv^	0.88	2.13	2.8678 (15)	141
O1*W*—H1*W*⋯O8	0.847 (18)	2.049 (18)	2.8812 (14)	167.4 (19)
O1*W*—H2*W*⋯O13^v^	0.861 (18)	2.233 (18)	3.0702 (14)	164.1 (19)

Symmetry codes: (i) 
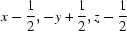
; (ii) 
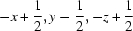
; (iii) 
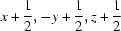
; (iv) 
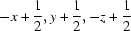
; (v) 
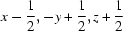
.

**Fig. 1.**
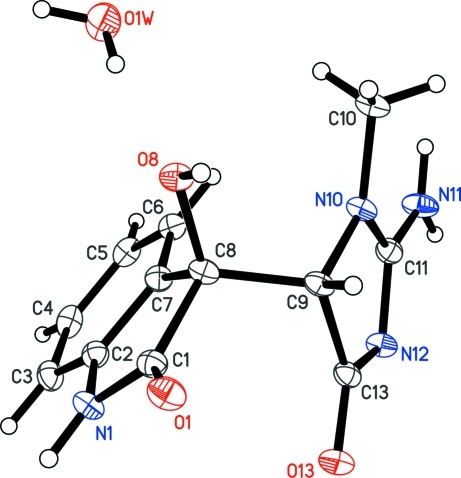
A view of the molecule with the atom numbering scheme. Displacement ellipsoids are drawn at the 50% probability level.

## supplementary crystallographic information

### Comment

In our endeavor to design and synthesize novel radiosensitizers such as (*Z*)-2-(*N*-benzylindol-3-ylmethylene)quinuclidin-3-one and (*Z*)-(±)-2-(*N*-benzylindol-3-ylmethylene)quinuclidin-3-ol derivatives (Sekhar *et al.*, 2003; Sonar *et al.*, 2007), we have undertaken the design, synthesis and structural analysis of a series of 3-(2-amino-1-methyl-4-oxo-4,5-dihydro-1*H*-imidazol-5-yl)-3- hydroxyindolin-2-one analogs with different substituents on the indole moiety. The primary goal for X-ray analysis of the title compound is to confirm the stereochemistry of the molecule and to obtain detailed information on the structural conformation that may be useful in structure–activity relationship (SAR) analysis. The title compound was prepared by the aldol condensation of indol-2,3-dione (isatin) with 2-amino-1-methyl-1*H*-imidazol-4(5*H*)-one (creatinine) in the presence of sodium acetate in acetic acid under microwave irradiation. The compound was crystallized from 2% aqueous glycol. This aldol condensation reaction proceeds by the formation of the *E*-enolate, as per the Zimmerman–Traxler model (Zimmerman & Traxler, 1957), which favors *anti* products, and leads to the formation of equimolar *RR* and *SS* enantiomers. The molecular structure and the atom-numbering scheme are shown in Fig. 1. The isatin ring is planar (r.m.s. deviation = 0.0112 (10) Å) with bond distances and angles comparable with those previously reported for other isatin derivatives (Frolova *et al.*, 1988; Usman *et al.*, 2002). Atoms C_8_ and C_9_ are the two chiral centers of the title compound. The X-ray studies revealed that the obtained compound is racemic (having equimolar *RR* and *SS* enantiomers). The indole and creatinine moieties make a dihedral angle of 56.75 (4)°. Intermolecular N—H···O and O—H···N hydrogen bonds stabilize the crystal structure and form a supramolecular architecture.

### Experimental

A mixture of isatin (1 mmol), creatinine (1.1 mmol) and sodium acetate (1.2 mmol) in acetic acid (1 ml) were irradiated in a domestic microwave oven for 40 sec with intermittent cooling every 10 sec. The reaction mixture was allowed to cool to room temperature, 10 ml of saturated sodium bicarbonate solution was added, and the mixture was stirred for 10 minutes. The precipitate thus obtained was collected by filtration, washed with cold water and dried, to afford the crude product. Crystallization from 2% aqueous glycol gave a light yellow crystalline product of 3-(2-amino-1-methyl-4-oxo-4,5-dihydro-1*H*- imidazol-5-yl)-3-hydroxyindolin-2-one monohydrate that was suitable for X-ray analysis. ^1^H NMR (DMSO-d_6_): δ 3.13 (*s*, 3H), 4.06 (*s*, 1H), 6.37 (*s*, 1H, OH), 6.73–6.75 (*d*, J = 7.5 Hz, 1H), 6.84–6.89 (*t*, J = 7.5 Hz, 1H), 7.04–7.06 (*d*, J = 7.5 Hz, 1H), 7.15–7.21 (*t*, J = 7.8 Hz, 1H), 7.51 (*bs*, 2H, NH_2_), 10.23 (*s*, 1H, NH) p.p.m.; ^13^C NMR (DMSO-d_6_): δ 32.59, 69.44, 76.28, 109.49, 121.1, 123.95, 127.98,129.34, 142.66, 171.76, 175.71, 182.26 p.p.m.

### Refinement

Non-water H atoms were found in difference Fourier maps and subsequently placed in idealized positions with constrained distances of 0.98 Å (RCH_3_), 1.00 Å (R_3_CH), 0.95 Å (C_Ar_H), 0.84 Å (O—H) and 0.88 Å (N—H) distances. U_iso_(H) values set to either 1.2U_eq_ or 1.5U_eq_(RCH_3_, OH) of the attached atom. The water H atoms were refined subject to distance and angle restraints and assigned U_iso_(H) values of 1.5U_eq_ of the water oxygen atom.

### Crystal data

**Table:**

C_12_H_12_N_4_O_3_·H_2_O	*F*(000) = 584
*M**_r_* = 278.27	*D*_x_ = 1.529 Mg m^−^^3^
Monoclinic, *P*2_1_/*n*	Cu *K*α radiation, λ = 1.54178 Å
Hall symbol: -P 2yn	Cell parameters from 9952 reflections
*a* = 8.3514 (1) Å	θ = 5.3–68.0°
*b* = 10.7166 (2) Å	µ = 0.99 mm^−^^1^
*c* = 13.9679 (2) Å	*T* = 90 K
β = 104.755 (1)°	Block, colourless
*V* = 1208.88 (3) Å^3^	0.20 × 0.15 × 0.06 mm
*Z* = 4	

### Data collection

**Table:**

Bruker X8 Proteum diffractometer	2181 independent reflections
Radiation source: fine-focus rotating anode	2121 reflections with *I* > 2σ(*I*)
graded multilayer optics	*R*_int_ = 0.035
Detector resolution: 5.6 pixels mm^-1^	θ_max_ = 68.0°, θ_min_ = 5.3°
φ and ω scans	*h* = −10→10
Absorption correction: multi-scan (*SADABS* in *APEX2*; Bruker, 2006)	*k* = −12→11
*T*_min_ = 0.780, *T*_max_ = 0.943	*l* = −16→16
17255 measured reflections	

### Refinement

**Table:**

Refinement on *F*^2^	Secondary atom site location: difference Fourier map
Least-squares matrix: full	Hydrogen site location: inferred from neighbouring sites
*R*[*F*^2^ > 2σ(*F*^2^)] = 0.034	H atoms treated by a mixture of independent and constrained refinement
*wR*(*F*^2^) = 0.088	*w* = 1/[σ^2^(*F*_o_^2^) + (0.0431*P*)^2^ + 0.777*P*] where *P* = (*F*_o_^2^ + 2*F*_c_^2^)/3
*S* = 1.04	(Δ/σ)_max_ < 0.001
2181 reflections	Δρ_max_ = 0.26 e Å^−^^3^
191 parameters	Δρ_min_ = −0.27 e Å^−^^3^
3 restraints	Extinction correction: *SHELXL97* (Sheldrick, 2008), Fc^*^=kFc[1+0.001xFc^2^λ^3^/sin(2θ)]^-1/4^
Primary atom site location: structure-invariant direct methods	Extinction coefficient: 0.0029 (7)

### Special details

**Table:**

Geometry. All e.s.d.'s (except the e.s.d. in the dihedral angle between two l.s. planes) are estimated using the full covariance matrix. The cell e.s.d.'s are taken into account individually in the estimation of e.s.d.'s in distances, angles and torsion angles; correlations between e.s.d.'s in cell parameters are only used when they are defined by crystal symmetry. An approximate (isotropic) treatment of cell e.s.d.'s is used for estimating e.s.d.'s involving l.s. planes.
Refinement. Refinement of *F*^2^ against ALL reflections. The weighted *R*-factor *wR* and goodness of fit *S* are based on *F*^2^, conventional *R*-factors *R* are based on *F*, with *F* set to zero for negative *F*^2^. The threshold expression of *F*^2^ > 2σ(*F*^2^) is used only for calculating *R*-factors(gt) *etc*. and is not relevant to the choice of reflections for refinement. *R*-factors based on *F*^2^ are statistically about twice as large as those based on *F*, and *R*- factors based on ALL data will be even larger.

### Fractional atomic coordinates and isotropic or equivalent isotropic displacement parameters (Å^2^)

**Table:**

	*x*	*y*	*z*	*U*_iso_*/*U*_eq_	
O1	−0.09392 (12)	0.10429 (9)	0.10391 (7)	0.0184 (2)	
N1	−0.15262 (14)	0.29911 (10)	0.15528 (8)	0.0158 (3)	
H1	−0.2381	0.3179	0.1060	0.019*	
C1	−0.06677 (16)	0.19095 (12)	0.16253 (9)	0.0146 (3)	
C2	−0.08770 (16)	0.37782 (12)	0.23678 (9)	0.0150 (3)	
C3	−0.14037 (16)	0.49625 (13)	0.25289 (10)	0.0183 (3)	
H3	−0.2307	0.5349	0.2072	0.022*	
C4	−0.05487 (17)	0.55663 (13)	0.33926 (10)	0.0192 (3)	
H4	−0.0875	0.6383	0.3527	0.023*	
C5	0.07679 (16)	0.49981 (13)	0.40589 (10)	0.0179 (3)	
H5	0.1332	0.5433	0.4640	0.021*	
C6	0.12728 (16)	0.37988 (12)	0.38869 (9)	0.0161 (3)	
H6	0.2163	0.3405	0.4348	0.019*	
C7	0.04456 (16)	0.31943 (12)	0.30268 (9)	0.0143 (3)	
O8	0.03982 (11)	0.09668 (8)	0.32447 (6)	0.0154 (2)	
H8	0.0481	0.0270	0.2986	0.023*	
C8	0.07032 (16)	0.19298 (12)	0.26163 (9)	0.0142 (3)	
C9	0.24325 (16)	0.17641 (12)	0.24175 (9)	0.0134 (3)	
H9	0.2513	0.0945	0.2090	0.016*	
C10	0.41472 (17)	0.10072 (12)	0.41347 (9)	0.0171 (3)	
H10A	0.5352	0.0920	0.4366	0.026*	
H10B	0.3655	0.0195	0.3909	0.026*	
H10C	0.3705	0.1309	0.4678	0.026*	
N10	0.37459 (13)	0.18921 (10)	0.33208 (8)	0.0138 (3)	
C11	0.45660 (16)	0.29528 (12)	0.32705 (9)	0.0148 (3)	
N11	0.57702 (14)	0.33716 (11)	0.40073 (8)	0.0195 (3)	
H11A	0.6067	0.2948	0.4564	0.023*	
H11B	0.6276	0.4075	0.3942	0.023*	
N12	0.40787 (14)	0.35577 (10)	0.23870 (8)	0.0156 (3)	
O13	0.21974 (12)	0.30271 (8)	0.09262 (7)	0.0174 (2)	
C13	0.28724 (16)	0.28445 (12)	0.18112 (9)	0.0140 (3)	
O1W	0.03730 (13)	0.14500 (11)	0.52700 (8)	0.0256 (3)	
H1W	0.023 (2)	0.1260 (19)	0.4666 (13)	0.038*	
H2W	−0.058 (2)	0.1453 (19)	0.5402 (14)	0.038*	

### Atomic displacement parameters (Å^2^)

**Table:**

	*U*^11^	*U*^22^	*U*^33^	*U*^12^	*U*^13^	*U*^23^
O1	0.0209 (5)	0.0161 (5)	0.0156 (5)	−0.0002 (4)	−0.0002 (4)	−0.0028 (4)
N1	0.0151 (5)	0.0148 (6)	0.0149 (5)	0.0014 (4)	−0.0010 (4)	0.0007 (4)
C1	0.0150 (6)	0.0141 (6)	0.0140 (6)	−0.0015 (5)	0.0025 (5)	0.0010 (5)
C2	0.0149 (6)	0.0147 (6)	0.0155 (6)	−0.0017 (5)	0.0043 (5)	0.0001 (5)
C3	0.0161 (6)	0.0153 (7)	0.0238 (7)	0.0021 (5)	0.0058 (5)	0.0026 (5)
C4	0.0205 (7)	0.0123 (6)	0.0278 (7)	−0.0005 (5)	0.0117 (6)	−0.0025 (5)
C5	0.0201 (7)	0.0169 (7)	0.0182 (7)	−0.0046 (5)	0.0079 (5)	−0.0044 (5)
C6	0.0170 (6)	0.0160 (7)	0.0153 (6)	−0.0013 (5)	0.0040 (5)	−0.0002 (5)
C7	0.0157 (6)	0.0120 (6)	0.0151 (6)	−0.0003 (5)	0.0038 (5)	0.0003 (5)
O8	0.0198 (5)	0.0106 (5)	0.0151 (5)	−0.0008 (4)	0.0032 (4)	0.0000 (3)
C8	0.0164 (7)	0.0116 (6)	0.0128 (6)	0.0004 (5)	0.0007 (5)	0.0007 (5)
C9	0.0155 (6)	0.0111 (6)	0.0115 (6)	0.0006 (5)	−0.0004 (5)	−0.0004 (5)
C10	0.0201 (7)	0.0139 (6)	0.0146 (6)	0.0003 (5)	−0.0006 (5)	0.0034 (5)
N10	0.0153 (5)	0.0113 (5)	0.0124 (5)	−0.0002 (4)	−0.0009 (4)	0.0013 (4)
C11	0.0156 (6)	0.0132 (6)	0.0147 (6)	0.0008 (5)	0.0026 (5)	−0.0001 (5)
N11	0.0228 (6)	0.0159 (6)	0.0156 (6)	−0.0059 (5)	−0.0029 (5)	0.0029 (4)
N12	0.0186 (6)	0.0129 (5)	0.0134 (5)	−0.0008 (4)	0.0006 (4)	0.0011 (4)
O13	0.0215 (5)	0.0154 (5)	0.0128 (5)	0.0004 (4)	−0.0003 (4)	0.0010 (3)
C13	0.0157 (6)	0.0116 (6)	0.0140 (6)	0.0030 (5)	0.0024 (5)	0.0000 (5)
O1W	0.0214 (5)	0.0353 (6)	0.0184 (5)	−0.0051 (4)	0.0019 (4)	−0.0057 (4)

### Geometric parameters (Å, °)

**Table:**

O1—C1	1.2206 (16)	C8—C9	1.5488 (18)
N1—C1	1.3531 (17)	C9—N10	1.4522 (16)
N1—C2	1.4096 (17)	C9—C13	1.5334 (17)
N1—H1	0.8800	C9—H9	1.0000
C1—C8	1.5557 (17)	C10—N10	1.4525 (16)
C2—C3	1.3803 (19)	C10—H10A	0.9800
C2—C7	1.3932 (18)	C10—H10B	0.9800
C3—C4	1.395 (2)	C10—H10C	0.9800
C3—H3	0.9500	N10—C11	1.3382 (17)
C4—C5	1.387 (2)	C11—N11	1.3209 (17)
C4—H4	0.9500	C11—N12	1.3612 (17)
C5—C6	1.3925 (19)	N11—H11A	0.8800
C5—H5	0.9500	N11—H11B	0.8800
C6—C7	1.3847 (18)	N12—C13	1.3540 (17)
C6—H6	0.9500	O13—C13	1.2366 (16)
C7—C8	1.5080 (17)	O1W—H1W	0.847 (18)
O8—C8	1.4192 (15)	O1W—H2W	0.861 (18)
O8—H8	0.8400		
			
C1—N1—C2	111.39 (11)	C7—C8—C1	101.94 (10)
C1—N1—H1	124.3	C9—C8—C1	110.33 (10)
C2—N1—H1	124.3	N10—C9—C13	100.04 (10)
O1—C1—N1	126.67 (12)	N10—C9—C8	111.46 (10)
O1—C1—C8	125.20 (11)	C13—C9—C8	112.21 (10)
N1—C1—C8	108.08 (10)	N10—C9—H9	110.9
C3—C2—C7	122.50 (12)	C13—C9—H9	110.9
C3—C2—N1	127.51 (12)	C8—C9—H9	110.9
C7—C2—N1	109.99 (11)	N10—C10—H10A	109.5
C2—C3—C4	116.96 (12)	N10—C10—H10B	109.5
C2—C3—H3	121.5	H10A—C10—H10B	109.5
C4—C3—H3	121.5	N10—C10—H10C	109.5
C5—C4—C3	121.36 (12)	H10A—C10—H10C	109.5
C5—C4—H4	119.3	H10B—C10—H10C	109.5
C3—C4—H4	119.3	C11—N10—C9	108.54 (10)
C4—C5—C6	120.82 (12)	C11—N10—C10	125.20 (11)
C4—C5—H5	119.6	C9—N10—C10	126.23 (10)
C6—C5—H5	119.6	N11—C11—N10	123.09 (12)
C7—C6—C5	118.39 (12)	N11—C11—N12	122.48 (12)
C7—C6—H6	120.8	N10—C11—N12	114.41 (11)
C5—C6—H6	120.8	C11—N11—H11A	120.0
C6—C7—C2	119.96 (12)	C11—N11—H11B	120.0
C6—C7—C8	131.47 (12)	H11A—N11—H11B	120.0
C2—C7—C8	108.56 (11)	C13—N12—C11	105.97 (11)
C8—O8—H8	109.5	O13—C13—N12	125.82 (12)
O8—C8—C7	110.67 (10)	O13—C13—C9	124.01 (11)
O8—C8—C9	110.48 (10)	N12—C13—C9	110.17 (10)
C7—C8—C9	113.64 (10)	H1W—O1W—H2W	108.3 (17)
O8—C8—C1	109.44 (10)		
			
C2—N1—C1—O1	179.49 (12)	O1—C1—C8—C9	59.80 (16)
C2—N1—C1—C8	1.73 (14)	N1—C1—C8—C9	−122.39 (11)
C1—N1—C2—C3	177.98 (13)	O8—C8—C9—N10	−64.03 (13)
C1—N1—C2—C7	−1.42 (15)	C7—C8—C9—N10	61.06 (14)
C7—C2—C3—C4	−0.05 (19)	C1—C8—C9—N10	174.83 (10)
N1—C2—C3—C4	−179.38 (12)	O8—C8—C9—C13	−175.30 (10)
C2—C3—C4—C5	−0.16 (19)	C7—C8—C9—C13	−50.22 (14)
C3—C4—C5—C6	−0.3 (2)	C1—C8—C9—C13	63.55 (13)
C4—C5—C6—C7	1.00 (19)	C13—C9—N10—C11	8.33 (13)
C5—C6—C7—C2	−1.19 (19)	C8—C9—N10—C11	−110.49 (12)
C5—C6—C7—C8	178.47 (13)	C13—C9—N10—C10	−169.97 (11)
C3—C2—C7—C6	0.7 (2)	C8—C9—N10—C10	71.21 (15)
N1—C2—C7—C6	−179.82 (11)	C9—N10—C11—N11	175.73 (12)
C3—C2—C7—C8	−179.00 (12)	C10—N10—C11—N11	−5.9 (2)
N1—C2—C7—C8	0.44 (14)	C9—N10—C11—N12	−5.46 (15)
C6—C7—C8—O8	64.51 (18)	C10—N10—C11—N12	172.86 (11)
C2—C7—C8—O8	−115.79 (12)	N11—C11—N12—C13	178.05 (12)
C6—C7—C8—C9	−60.47 (18)	N10—C11—N12—C13	−0.77 (15)
C2—C7—C8—C9	119.23 (12)	C11—N12—C13—O13	−174.16 (12)
C6—C7—C8—C1	−179.17 (13)	C11—N12—C13—C9	6.41 (14)
C2—C7—C8—C1	0.53 (13)	N10—C9—C13—O13	171.44 (12)
O1—C1—C8—O8	−61.96 (16)	C8—C9—C13—O13	−70.29 (16)
N1—C1—C8—O8	115.85 (11)	N10—C9—C13—N12	−9.12 (13)
O1—C1—C8—C7	−179.17 (12)	C8—C9—C13—N12	109.15 (12)
N1—C1—C8—C7	−1.36 (13)		

### Hydrogen-bond geometry (Å, °)

**Table:**

*D*—H···*A*	*D*—H	H···*A*	*D*···*A*	*D*—H···*A*
N1—H1···O1W^i^	0.88	1.96	2.8128 (15)	163
O8—H8···N12^ii^	0.84	1.97	2.7984 (14)	170
N11—H11A···O13^iii^	0.88	2.17	3.0371 (15)	171
N11—H11B···O1^iv^	0.88	2.13	2.8678 (15)	141
O1W—H1W···O8	0.85 (2)	2.05 (2)	2.8812 (14)	167 (2)
O1W—H2W···O13^v^	0.86 (2)	2.23 (2)	3.0702 (14)	164 (2)

Symmetry codes: (i) *x*−1/2, −*y*+1/2, *z*−1/2; (ii) −*x*+1/2, *y*−1/2, −*z*+1/2; (iii) *x*+1/2, −*y*+1/2, *z*+1/2; (iv) −*x*+1/2, *y*+1/2, −*z*+1/2; (v) *x*−1/2, −*y*+1/2, *z*+1/2.
